# The social, physical and economic impact of lymphedema and hydrocele: a matched cross-sectional study in rural Nigeria

**DOI:** 10.1186/s12879-019-3959-6

**Published:** 2019-04-23

**Authors:** Obiora A. Eneanya, Tini Garske, Christl A. Donnelly

**Affiliations:** 1 MRC Centre for Global Infectious Disease Analysis, Department of Infectious Disease Epidemiology, Imperial College London, Faculty of Medicine, Norfolk Place, London, W2 1PG UK; 2 Department of Statistics, University of Oxford, Oxford, UK

**Keywords:** Lymphatic filariasis, Lymphedema, Hydrocele, Psychosocial impacts, Mental health

## Abstract

**Background:**

Lymphatic filariasis (LF) is a mosquito-borne parasitic disease and a major cause of disability worldwide. To effectively plan morbidity management programmes, it is important to estimate disease burden and evaluate the needs of patients. This study aimed to estimate patient numbers and characterise the physical, social and economic impact of LF in in rural Nigeria.

**Methods:**

This is a matched cross-sectional study which identified lymphedema and hydrocele patients with the help of district health officers and community-directed distributors of mass drug administration programmes. A total of 52 cases were identified and matched to 52 apparently disease-free controls, selected from the same communities and matched by age and sex. Questionnaires and narrative interviews were used to characterise the physical, social and economic impact of lymphedema and hydrocele.

**Results:**

Forty-eight cases with various stages of lower limb lymphedema, and 4 with hydrocele were identified. 40% of all cases reported feeling stigma and were 36 times (95% CI: 5.18–1564.69) more likely to avoid forms of social participation. Although most cases engaged in some form of income-generating activity, these were low paid employment, and on average cases spent significantly less time than controls working. The economic effects of lower income were exacerbated by increased healthcare spending, as cases were 86 times (95% CI: 17.48–874.90) more likely to spend over US $125 on their last healthcare payment.

**Conclusion:**

This study highlights the importance of patient-search as a means of estimating the burden of LF morbidity in rural settings. Findings from this work also confirm that LF causes considerable psychosocial and economic suffering, all of which adversely affect the mental health of patients. It is therefore important to incorporate mental health care as a major component of morbidity management programmes.

**Electronic supplementary material:**

The online version of this article (10.1186/s12879-019-3959-6) contains supplementary material, which is available to authorized users.

## Background

Lymphedema, hydrocele and acute inflammatory episodes (adenolymphangitis (ADL)), are the most common clinical symptoms of lymphatic filariasis (LF) [1]. It is estimated that globally 15 million people suffer from lymphedema and 25 million men from hydrocele [2]. One of the main goals of the Global Programme for Elimination of Lymphatic filariasis (GPELF) is to manage morbidity and prevent disability among people already infected [3].

Previous studies have shown that lymphedema reduces the ability of individuals to perform basic daily activities independently [4–10]. This incapacitation could lead to reduced work hours and increased healthcare spending. Treatment costs deplete financial resources and LF patients are sometimes considered to be financial burdens by their families. LF patients with advanced stage lymphedema (elephantiasis) and hydrocele perceive stigma from community and members of their families: in many instances this results in abandonment by their spouse, the inability to get married or the interruption of education [8]. This could lead to anxiety and have adverse effects on mental health.

Studies on the social aspects of LF in Nigeria mainly focus on knowledge, attitudes and local perceptions of disease [7, 11, 12]. Lymphedema and hydrocele are well known among members of prevalent communities and in many instances are recognised by local terminologies; however, the perceived causes are often reported to be spiritual, hereditary, poor hygiene and sanitation and engaging in strenous physical activities such as walking for long distances [7, 11–13]. It is thought that improved knowledge and understanding of LF will result in greater adherence to chemotherapy and best practices in morbidity management, discouraging traditional treatment practices which could harm the patients [2].

Although most endemic districts in Nigeria have undergone multiple rounds of mass drug administration (MDA) [14], transmission is yet to be interrupted in all but two states [15]. However, the national LF programme has projected that implementation of MDA will stop in 2020 [16], in line with the goal set by the GPELF [17]. This will shift programmatic attention more towards morbidity management. The integration of treatment and morbidity management programmes has been found to be more effective than either programme being implemented alone [18]. It has also been shown that morbidity management programmes raise awareness about disease in targeted communities thus enhancing adherence to MDA [2]. It will therefore be beneficial to start morbidity management as soon as resources are available. Accurate estimates of disease burden and understanding patient requirements will be useful for implementing morbidity management programmes in endemic communities. This study aims to characterise LF impacts to better prioritise morbidity management practices in endemic areas and to suggest a structure for patient reporting systems.

## Methods

### Description of study location

**Fig. 1:**
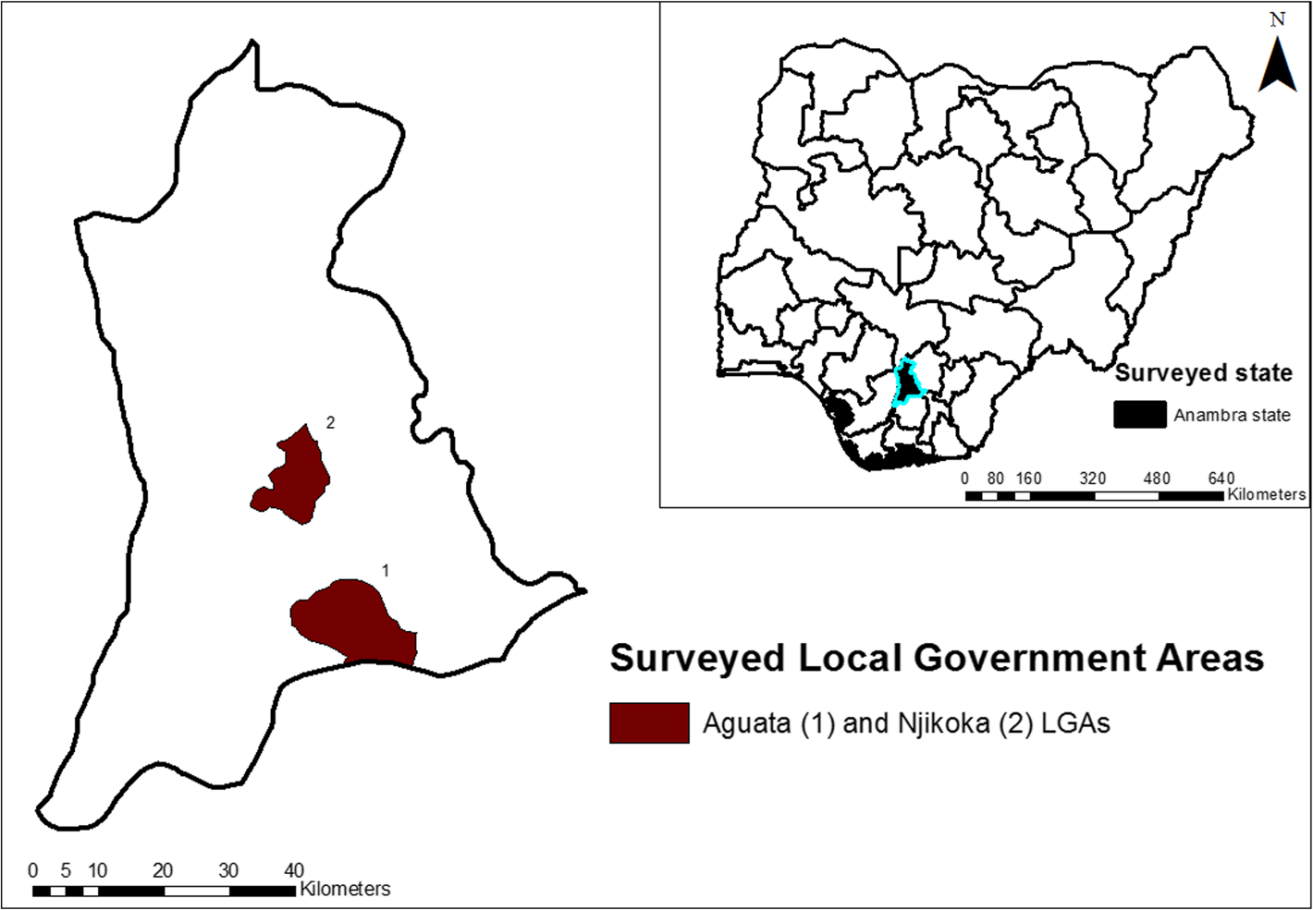
Map of Anambra state, with surveyed LGAs highlighted. Inset is a map of Nigeria, highlighting Anambra state. (Map was created by Obiora A. Eneanya)

As shown in Fig. 1, this study was conducted in Aguata (5.94 N, 7.05E) and Njikoka (6.17 N, 6.96E) Local Government Areas (LGAs) of Anambra State, south-east Nigeria. Anambra State has average day and night temperatures of 28.9 °C and 19.3 °C respectively [16], and is of a tropical forest vegetation. The region has two distinct seasons, rainy and dry seasons. The rainy season typically runs from March to October, peaking in June with rainfall estimates of around 1866 mm annually. The study area has a combined population of 517,000 and contains 19 towns, most of which are rural. The predominant occupations of inhabitants are farmers, artisans, traders/small business owners and low-cadre government workers. Primary health centres serve as an important source of medical care, offering treatment for minor ailments whilst also providing maternity services to surrounding villages. Inhabitants of these communities also seek healthcare from traditional and faith-based centres. Mapping surveys in the study area conducted in 2008, prior to the commencement of MDA programmes, recorded prevalence levels of 28 and 29% in Aguata and Njikoka LGAs, respectively [16].

### Participant identification

As there was no previous listing of LF cases, we identified cases using the already established infrastructure for MDA including community-directed distributors, community health workers and local volunteers in the study area. A network of local informants, town criers and religious leaders was established and helped identify additional cases within the communities. Our network for patient identification was briefed on the study objectives, need for patient anonymity and all other ethical limits of the study. Potential cases were asked to contact a member of the district health team, who upon obtaining written informed consent, invited the interviewer to review the candidate and conduct an interview when lymphedema/hydrocele was confirmed. Confirmed cases were asked whether they knew of any member of the village or community who had the same condition. When a referral was made, candidates were contacted and invited to take part in the study if confirmed to have lymphedema or hydrocele.

### Interviews

Most of the interviews for cases were conducted in the district health centres. For cases who were unable to travel to the nearest health centre due to severity of lymphedema and/or financial constraints, interviews were conducted at their residences.

Interviews were conducted in a private room with only the interviewer and participant present. This measure afforded the participants an atmosphere of confidentiality, encouraging candid responses to questions. The objectives of the study and expected benefits were explained to all participants and written consent was obtained before every interview. All participants were informed of LF preventive measures and, as MDA is still ongoing in the communities, were advised to adhere to the recommended treatment regimen.

Unlike for the cases, where most interviews were conducted in the health centres, interviews for controls were conducted within the communities. In surveyed villages, the interviewer determined an approximate centre of the village, which is usually the location of the village hall (a community centre). By spinning a pen [19], a random direction was chosen and every second building on that street was selected for the survey. Only one participant in each household was interviewed. Here, a ‘household’ was defined as a nuclear family. Where there were more than one household living in a building, only one adult was allowed to participate in the study. In instances where cases were interviewed in their homes, an adult in the building directly opposite or adjacent was selected as control. In instances where matching controls were not in the immediate opposite or adjacent buildings, the data collector sampled the nearest house with a suitable matching control.

### Recruitment and matching criteria

All participants were required to have resided in the community for at least 10 years preceding the date of interview. Controls were recruited from the same population as cases, and matched by age, sex and residential location (Additional file 1: Table S1). As some participants were unsure of their exact date of birth, matching age was defined as within a 5-year age bracket. This was more easily ascertained as most communities have an established age grade system, where people born within 5 years of each other are grouped into same age grade and took part in activities such as community policing.

### Survey instrument

The survey tool used to characterise the impact of lymphedema was adopted from Martindale et al. [4]. This was originally adapted from the Standard European Quality of Life (EuroQol) [20] project, and had been validated in LF studies [21, 22]. We expanded the instrument to include questions on treatment-seeking behaviour, stigma and self-esteem, relationship with spouse, marriageability, work hours, healthcare expenditure, and bed net usage. Questions for stigma assessment had been validated in Nigeria and across other rural African settings for onchocercal skin disease (OSD) [23]. We adopted these validated questions for our study because LF presents with somewhat similar challenges with stigma, relationships and marriageability as OSD. To characterise other socioeconomic impacts of LF we included questions on occupation, educational qualification, marital status and treatment cost. These had been previously used in similar studies elsewhere [5, 9]. Data were collected in structured interviews and questions were asked in English; however for the benefit of participants who were not fluent in oral or written English, the questions were explained in their local language, *Igbo*. The translated questions were pre-tested on a selection of local residents and healthcare workers at the district health centre to ensure that questions were properly phrased and the main message was not lost in translation.

### Assessment of lymphedema

The severity of (limb) lymphedema was assessed using the Dreyer staging guidelines [1], based on reversibility of swelling, presence of skin folds, knobs, bumps, and lumps. Scores for the severity of lymphedema range from 1 to 7, score 1 being the least severe and score 7 being the most severe. Table 1 outlines the characteristics of each Dreyer stage score.

**Table 1:** Clinical characteristics of Dreyer stages used for grading severity of lymphedema [1]

Dreyer stage	Clinical characteristics
1	Swelling is reversible overnight
2	Swelling is NOT reversible overnight
3	Presence of shallow skin folds
4	Presence of knobs/bumps/lumps
5	Presence of deep skin folds
6	Presence of ‘mossy foot’ and bad odour
7	Unable to care for self and perform daily activities

### Statistical and qualitative analysis

Age was stratified by 10-year age groups and ranged from 21 years to > 60 years. Participants who were currently single at the time of interview were classified as either ‘never married’ or ‘divorced’. Participants who held government jobs or other forms of salaried employment that required either secondary school education or specialised training were categorised as ‘skilled’. Those who engaged in petty trading and daily paid artisans such as butchers/meat-sellers and labourers were categorised as ‘unskilled’. For treatment-seeking behaviour, untrained care practitioners administering treatment with locally made herbs were categorised as traditional healers, whereas spiritual healers are those who seek divine healing, and carry out their activities in churches and prayer temples.

Descriptive statistics of cases and controls were used to summarize demographic, physical and socioeconomic characteristics (Additional file 2: Table S2). Fisher’s exact tests were used to test for significant associations between LF (and among cases LF severity) and physical and socioeconomic characteristics as well as treatment-seeking behaviour. The conditional maximum likelihood estimates of the odds ratios given. Sign tests were used to test for within-matched-pair differences. All quantitative data were analysed in R (v.3.3.2) [24].

Self-care, mobility, ability to engage in usual activity, social participation, pain frequency and severity, cognition, anxiety, and sleep problems were used to assess the psychosocial impacts of lymphedema and hydrocele, whereas work hours, occupation and treatment expenditure were used to characterise the economic impact.

Narrative responses were coded into dominant themes. Similar themes were grouped together and summarized. These open-ended responses were used to give participants a chance to elaborate on the impacts of LF in their own words, rather than answering only to set questions in structured interviews. The main purpose of this was to elaborate on responses from the structured interviews and better understand the psychosocial and economic impacts of LF morbidity, perhaps finding relationships which structured interviews might have missed. Some direct quotes are included in this work to give an idea of the experiences of chronic LF patients.

## Results

### Quantitative analysis

Table 2 shows *p*-values from Fisher’s exact test with conditional maximum likelihood estimates of odds ratios for factors associated with lymphedema and hydrocele. Survey participants were 85% (95% CI: 38–97%) less likely to have skilled or salaried employment. Although most patients engaged in various forms of income-generating activities, there were mostly low paid and unskilled employment, as they do not have adequate educational qualification to take up skilled employment. Lymphedema and hydrocele were also major contributing factors to social participation, as cases were 36.05 times (95% CI: 5.18–1564.69) more likely to totally avoid various forms of social engagements and activities. Lymphedema and hydrocele appeared to limit work hours per day, as cases were 13 times (95% CI: 1.60–589.43) more likely to work for between 1 to 4 h per day. Cases were found to be 85.90 times (95% CI: 17.48–874.90) more likely to spend over 40,000 Naira (about US $125) on their last healthcare expenditure. Also, cases were 72% (95% CI: 29–89%) less likely to use bed nets. Social characteristics such as self-care, mobility, activity levels, pain/discomfort, pain frequency, pain severity and anxiety all show strong associations with lymphedema and hydrocele.

**Table 2:** Factors associated with lymphedema and hydrocele

Characteristics	Control (*N* = 52)	Cases (N = 52)	OR (95%CI)	*P*-value (Fisher’s exact)
Marital status				
Married	52 (100%)	40 (76.92%)	1.00	
Divorced	0 (0%)	2 (3.85%)	Inf (0.233 – Inf)	0.197
Never married	0 (0%)	10 (19.23%)	**Inf (2.68 – Inf)**	**< 0.001**
Occupational level				
Unskilled	31 (59.61%)	44 (84.61%)	1.00	
No work	7 (13.46%)	5 (9.62%)	0.51 (0.12–2.05)	0.351
Skilled	14 (26.92%)	3 (5.77%)	**0.15 (0.03–0.62)**	**0.002**
Educational level				
Primary education	18 (34.62%)	26 (50%)	1.00	
No formal education	1 (1.92%)	9 (17.31%)	6.07 (0.73–287.58)	0.079
Secondary education	18 (34.61%)	13 (25%)	0.50 (0.17–1.40)	0.165
Tertiary education	13 (25%)	4 (7.69%)	**0.22 (0.04–0.86)**	**0.021**
Post graduate education	2 (3.84%)	0 (0%)	0 (0.00–4.04)	0.184
Mobility				
No problem	52 (100%)	20 (38.46%)	1.00	
Mild problem	0 (0%)	13 (25.00%)	**Inf (6.89 – Inf)**	**< 0.001**
Moderate problem	0 (0%)	3 (5.77%)	**Inf (0.98 – Inf)**	**0.026**
Severe problem	0 (0%)	16 (30.77%)	**Inf (8.69 – Inf)**	**< 0.001**
Self-care				
No problem	52 (100%)	35 (67%)	1.00	
Difficulty in self-care	0 (0%)	7 (13.46%)	**Inf (1.97 – Inf)**	**< 0.001**
Only essential needs are met	0 (0%)	3 (5.77%)	**Inf (0.58 – Inf)**	**0.072**
Require someone to help for care	0 (0%)	6 (11.54%)	**Inf (1.61 – Inf)**	**0.006**
Unable to take care of self	0 (0%)	1 (1.92%)	Inf (0.37 – Inf)	0.41
Usual activity				
No problem	52 (100%)	38 (73.08%)	1.00	
Usual activity performed with difficulty	0 (0%)	9 (17.31%)	**Inf (2.48 – Inf)**	**0.001**
Only essential activities performed	0 (0%)	4 (7.69%)	**Inf (0.85 – Inf)**	**0.037**
No activity at all	0 (0%)	1 (1.92%)	Inf (0.34 – Inf)	0.429
Pain and discomfort				
No pain	40 (76.92%)	1 (1.92%)	1.00	
Mild pain, without self-treatment	9 (17.31%)	20 (38.46%)	**81.27 (10.58–3678.40)**	**< 0.001**
Mild pain, with self-treatment	1 (1.92%)	7 (13.46%)	**174.16 (11.83–11,241.6)**	**< 0.001**
Compelled to rest due to pain	0 (0%)	24 (46.15%)	**Inf (75.48 – Inf)**	**< 0.001**
Consult doctor and total rest	2 (3.85%)	0 (0%)	0 (0.00–789.64)	1
Pain frequency				
No pain	40 (76.92%)	1 (1.92%)	1.00	
Weekly	2 (3.85%)	14 (26.92%)	**133.14 (11.72–7481.31)**	**< 0.001**
Monthly	2 (3.85%)	9 (17.31%)	**108.53 (14.14–4876.50)**	**< 0.001**
Daily	8 (15.38%)	24 (46.15%)	**206.92 (19.38–10,808.72)**	**< 0.001**
Constant	0 (0%)	4 (7.69%)	**Inf (9.55 – Inf)**	**< 0.001**
Pain severity				
No pain	40 (76.92%)	1 (1.92%)	1.00	
Mild	9 (17.31%)	3 (5.77%)	12.44 (0.89–713.07)	0.032
Medium	1 (1.92%)	18 (34.61%)	**434.47 (34.52–16,384.00)**	**< 0.001**
Severe	2 (3.85%)	30 (57.69%)	**439.78 (43.96–16,384.00)**	**< 0.001**
Cognition				
No problem	52 (100%)	46 (88.46%)	1.00	
Reduced concentration, memory affected	0 (0%)	4 (7.69%)	Inf (0.70 – Inf)	0.054
Loss of concentration	0 (0%)	0 (0%)	Inf (0.20 – Inf)	0.228
Anxiety				
No anxiety	52 (100%)	6 (11.54%)	1.00	
Does not interfere with performance	0 (0%)	9 (17.31%)	**Inf (12.27 – Inf)**	**< 0.001**
Leads to low performance and irritating tendency	0 (0%)	22 (42.31%)	**Inf (33.17 – Inf)**	**< 0.001**
No performance and total detachment	0 (0%)	13 (25%)	**Inf (18.69 – Inf)**	**< 0.001**
Suicidal tendency	0 (0%)	2 (3.85%)	**Inf (1.31 – Inf)**	**0.016**
Sleep problems				
No sleep problem	42 (76.92%)	22 (42.31%)	1.00	
Sleep problem rarely	10 (23.08%)	9 (17.31%)	1.7 (0.53–5.48)	0.418
Sleep problem occasionally	0 (0%)	8 (15.39%)	**Inf (2.87 – Inf)**	**0.001**
Sleep problem often	0 (0%)	6 (11.53%)	**Inf (2.00 – Inf)**	**0.003**
Cannot sleep most nights	0 (0%)	7 (13.46%)	**Inf (2.43 – Inf)**	**0.001**
Social participation				
No problem with social participation	51 (98.08%)	26 (50%)	1.00	
Avoid social activities as far as possible	1 (1.92%)	19 (36.54%)	**36.05 (5.18–1564.69)**	**< 0.001**
Total avoidance of social activities	0 (0%)	7 (13.46%)	**Inf (2.55 – Inf)**	**0.001**
Work hours				
> 8 h	24 (46.15%)	20 (38.46%)	1.00	
5–8 h	9 (17.31%)	5 (9.62%)	0.67 (0.15–2.9 - 68)	0.556
1–4 h	1 (1.92%)	11 (21.15%)	**12.70 (1.60–589.43)**	**0.007**
No work	7 (13.46%)	5 (9.62%)	0.86 (0.18–3.72)	1
Seasonal work	6 (11.54%)	2 (3.85%)	0.41 (0.04–2.61)	0.442
Retired	5 (9.62%)	9 (17.31%)	2.13 (0.54–9.50)	0.358
Treatment expenditure on last hospital visit				
< 40,000 Naira (about US $125)	48 (92.31%)	9 (17.31%)	1.00	
> 40,000 Naira (about US $125)	2 (3.85%)	35 (67.31%)	**85.90 (17.48–874.90)**	**< 0.001**
Unable to quantify	2 (3.85%)	8 (15.39%)	**19.90 (3.28–221.68)**	**< 0.001**
Bed net/door and window screens				
No net use	18 (34.62%)	30 (57.69%)	1.00	
Screen on doors and windows	2 (3.85%)	7 (13.46%)	2.07 (0.34–22.61)	0.471
Bed net use	32 (61.54%)	15 (28.85%)	**0.28 (0.11–0.71)**	**0.004**

Reference category – that with the highest count was chosen as the reference category

Results at 0.05 significance level appear in bold

To test the robustness of the results, matched analyses were undertaken using sign tests in which covariates were scored as integers in the order presented in Table 3 (for example: “No work” = 1, “Unskilled” = 2, “Skilled” = 3). Differences were calculated within each matched pair. If there were no systematic differences, then the proportion of negative (case minus control) differences would not be significantly different from 0.5 (50%). Cases were significantly more likely to have lower level of education, mobility difficulties, reduced activity, more pain and discomfort, more frequent and severe pain, reduced concentration, more sleep problems, more anxiety, less social participation, more healthcare expenditure and be divorced or never married than their matched controls. These have been highlighted in boldface.

**Table 3:** Matched analysis of factors associated with lymphedema and hydrocele in study participants tested using sign tests

Characteristics	Control (N = 52)	Cases (N = 52)	Number positive differences (%)	Number negative differences (%)	Proportion of negative differences (Exact binomial CI)
Marital status			0 (0%)	12 (100%)	**1 (0.74–1.00)**
Never married	0 (0%)	10 (19.23%)			
Divorced	0 (0%)	2 (3.85%)			
Married	52 (100%)	40 (76.92%)			
Occupational level			8 (33.33%)	16 (66.67%)	0.67 (0.45–0.84)
No work	7 (13.46%)	5 (9.62%)			
Unskilled	31 (59.61%)	44 (84.61%)			
Skilled	14 (26.92%)	3 (5.77%)			
Educational level			9 (24.32%)	28 (75.68%)	**0.76 (0.59–0.88)**
No formal education	1 (1.92%)	9 (17.31%)			
Primary education	18 (34.62%)	26 (50%)			
Secondary education	18 (34.61%)	13 (25%)			
Tertiary education	13 (25%)	4 (7.69%)			
Post graduate education	2 (3.84%)	0 (0%)			
Mobility			32 (100.00%)	0 (0.00%)	**0 (0–0.11)**
No problem	52 (100%)	20 (38.46%)			
Mild problem	0 (0%)	13 (25.00%)			
Moderate problem	0 (0%)	3 (5.77%)			
Severe problem	0 (0%)	16 (30.77%)			
Self-care			17 (100.00%)	0 (0.00%)	**0 (0–0.20)**
No problem	52 (100%)	35 (67%)			
Difficulty in self-care	0 (0%)	7 (13.46%)			
Only essential needs are met	0 (0%)	3 (5.77%)			
Require someone to help for care	0 (0%)	6 (11.54%)			
Unable to take care of self	0 (0%)	1 (1.92%)			
Usual activity			14 (100.00%)	0 (0.00%)	**0 (0–0.23)**
No problem	52 (100%)	38 (73.08%)			
Usual activity performed with difficulty	0 (0%)	9 (17.31%)			
Only essential activities performed	0 (0%)	4 (7.69%)			
No activity at all	0 (0%)	1 (1.92%)			
Pain and discomfort			45 (93.75%)	3 (6.25%)	**0.06 (0.01–0.17)**
No pain	40 (76.92%)	1 (1.92%)			
Mild pain, without self-treatment	9 (17.31%)	20 (38.46%)			
Mild pain, with self-treatment	1 (1.92%)	7 (13.46%)			
Compelled to rest due to pain	0 (0%)	24 (46.15%)			
Consult doctor and total rest	2 (3.85%)	0 (0%)			
Pain frequency			43 (91.49%)	4 (8.51%)	**0.085 (0.02–0.2)**
No pain	40 (76.92%)	1 (1.92%)			
Weekly	2 (3.85%)	14 (26.92%)			
Monthly	2 (3.85%)	9 (17.31%)			
Daily	8 (15.38%)	24 (46.15%)			
Constant	0 (0%)	4 (7.69%)			
Pain severity			48 (100.00%)	0 (0.00%)	**0 (0–0.07)**
No pain	40 (76.92%)	1 (1.92%)			
Mild	9 (17.31%)	3 (5.77%)			
Medium	1 (1.92%)	18 (34.61%)			
Severe	2 (3.85%)	30 (57.69%)			
Cognition			6 (100.00%)	0 (0.00%)	**0 (0–0.46)**
No problem	52 (100%)	46 (88.46%)			
Reduced concentration, memory affected	0 (0%)	4 (7.69%)			
Loss of concentration	0 (0%)	2 (3.85%)			
Anxiety			46 (100.00%)	0 (0.00%)	**0 (0–0.08)**
No anxiety	52 (100%)	6 (11.54%)			
Does not interfere with performance	0 (0%)	9 (17.31%)			
Leads to low performance and irritating tendency	0 (0%)	22 (42.31%)			
No performance and total detachment	0 (0%)	13 (25%)			
Suicidal tendency	0 (0%)	2 (3.85%)			
Sleep problems			28 (87.50%)	4 (12.50%)	**0.13 (0.04–0.29)**
No sleep problem	42 (76.92%)	22 (42.31%)			
Sleep problem rarely	10 (23.08%)	9 (17.31%)			
Sleep problem occasionally	0 (0%)	8 (15.39%)			
Sleep problem often	0 (0%)	6 (11.53%)			
Cannot sleep most nights	0 (0%)	7 (13.46%)			
Social participation			26 (96.30%)	1 (3.70%)	**0.04 (0.0009–0.19)**
No problem with social participation	51 (98.08%)	26 (50%)			
Avoid social activities as far as possible	1 (1.92%)	19 (36.54%)			
Total avoidance of social activities	0 (0%)	7 (13.46%)			
Work hours			19 (44.19%)	24 (55.81%)	0.56 (0.40–0.71)
No work	7 (13.46%)	5 (9.62%)			
Retired	5 (9.62%)	9 (17.31%)			
Seasonal work	6 (11.54%)	2 (3.85%)			
1–4 h	1 (1.92%)	11 (21.15%)			
5–8 h	9 (17.31%)	5 (9.62%)			
> 8 h	24 (46.15%)	20 (38.46%)			
Treatment expenditure on last hospital visit			33 (97.06%)	1 (2.94%)	**0.03 (0.0007–0.15)**
< 40,000 Naira (about US $125)	40 (95.24%)	9 (21.43%)			
> 40,000 Naira (about US $125)	2 (4.76%)	33 (78.57%)			
Bed net/door and window screens			7 (22.58%)	24 (77.42%)	**0.77 (0.59–0.90)**
No net use	18 (34.62%)	30 (57.69%)			
Screen on doors and windows	2 (3.85%)	7 (13.46%)			
Bed net use	32 (61.54%)	15 (28.85%)			

Table 4 describes the factors associated with lymphedema and hydrocele according to severity of disease. Here severity was categorised into low and high. Dreyer stage 1–5 were considered low severity, while Dreyer stage 6–7 and hydrocele were considered high severity. 69.2% of cases were in the low severity category. Cases with high severity of LF were about 14 times (95% CI: 2.14–169.67) more likely to perform usual activity with difficulty, and 10 times (95% CI: 2.16–54.24) more likely to have sleep problems. Higher severity of disease was also associated with cognition difficulties and anxiety.

**Table 4:** Factors associated to lymphedema and hydrocele according to severity of disease

Characteristics	Low severity (*N* = 36)	High severity (*N* = 16)	OR (95%CI)	*P*-value
Sex				
Male	25 (48.08%)	11 (21.15%)	1	
Female	11 (25.15%)	5 (9.62%)	1.03 (0.23–4.27)	1
LGA				
Aguata	18 (34. 62%)	12 (23.08%)	1	
Njikoka	18 (34.62%)	4 (7.69%)	1.55 (0.40–6.05)	0.558
Age				
21–30	3 (5.77%)	0 (0.00%)	1	
31–40	4 (7.69%)	4 (7.69%)	Inf (0.24 – Inf)	0.236
41–50	9 (17.31%)	5 (9.62%)	Inf (0.17 – Inf)	0.514
51–60	8 (15.38%)	3 (5.77%)	Inf (0.10 – Inf)	1
> 60	12 (23.08%)	4 (7.69%)	Inf (0.10 – Inf)	1
Marital status				
Married	27 (51.92%)	13 (25.00%)	1	
Divorced	2 (3.85)	0 (0.00%)	0 (0.00–12.07)	1
Never married	7 (13.46%)	3 (5.77)	0.89 (0.13–4.76)	1
Occupational level				
Unskilled	28 (53.85%)	16 (30.77%)	1	
No work	5 (9.62%)	0 (0.00%)	0 (0.00–2.19)	0.158
Skilled	3 (5.77%)	0 (0.00%)	0 (0.00–4.70)	0.541
Educational level				
Primary education	20 (38.46%)	6 (11.54%)	1	
No formal education	6 (11.54%)	3 (5.77%)	1.64 (0.20–11.10)	0.665
Secondary education	7 (13.46%)	6 (11.54%)	2.77 (0.54–14.77)	0.164
Tertiary education	3 (5.77%)	1 (1.92%)	1.11 (0.02–17.13)	1
Mobility				
No problem	17 (32.69%)	3 (5.77%)	1	
Mild problem	8 (15.38%)	5 (9.62%)	3.40 (0.51–27.64)	0.213
Moderate problem	2 (3.85%)	1 (1.92%)	2.67 (0.04–69.42)	0.453
Severe problem	9 (17.31%)	7 (13.46%)	4.22 (0.74–31.62)	0.073
Self-care				
No problem	28 (53.85%)	7 (13.46%)	1	
Difficulty in self-care	3 (5.77%)	4 (7.69%)	5.07 (0.69–43.25)	0.063
Only essential needs are met	2 (3.85%)	1 (1.92%)	1.96 (0.03–42.95)	0.519
Require someone to help for care	2 (3.85%)	4 (7.69%)	7.48 (0.88–98.74)	0.035
Unable to take care of self	1 (1.92%)	0 (0.00%)	0 (0.00–161.18)	1
Usual activity				
No problem	31 (59.62%)	7 (13.46%)	1	
Usual activity performed with difficulty	2 (3.85%)	7 (13.46%)	**14.30 (2.14–169.67)**	**0.001**
Only essential activities performed	3 (5.77%)	1 (1.92%)	1.46 (0.02–21.68)	1
No activity at all	0 (0.00%)	1 (1.92%)	Inf (0.10 – Inf)	0.205
Pain and discomfort				
No pain	0 (0.00%)	1 (1.92%)	1	
Mild pain, without self-treatment	18 (34.62%)	2 (3.85%)	0 (0.00–6.50)	0.143
Mild pain, with self-treatment	3 (5.77%)	4 (7.69%)	0 (0.00–64.94)	1
Compelled to rest due to pain	15 (28.85%)	9 (17.31%)	0 (0.00–26.00)	0.4
Pain frequency				
No pain	0 (0.00%)	1 (1.92%)	1	
Weekly	5 (9.62%)	4 (7.69%)	0 (0.00–39.00)	1
Monthly	19 (36.54%)	5 (9.62%)	0 (0.00–12.31)	0.24
Daily	10 (19.23%)	4 (7.69%)	0 (0.00–19.50)	0.333
Constant	2 (3.85%)	2 (3.85%)	0 (0.00–58.45)	1
Pain severity				
No pain	0 (0.00%)	1 (1.92%)	1	
Mild	3 (5.77%)	0 (0.00%)	0 (0.00–13.00)	0.250
Medium	16 (30.77%)	2 (3.85%)	0 (0.00–7.31)	0.158
Severe	17 (32.69%)	13 (25.00%)	0 (0.00–32.12)	0.452
Cognition				
No problem	36 (69.23%)	10 (19.23%)	1	
Reduced concentration, memory affected	0 (0.00%)	4 (7.69%)	**Inf (1.96 – Inf)**	**0.004**
Loss of concentration	0 (0.00%)	2 (3.85%)	Inf (0.59 – Inf)	0.059
Anxiety				
No anxiety	6 (11.54%)	0 (0.00%)	1	
Does not interfere with performance	9 (17.31%)	0 (0.00%)	0 (0.00 – Inf)	1
Leads to low performance and irritating tendency	19 (36.54%)	3 (5.77%)	Inf (0.11 – Inf)	1
No performance and total detachment	2 (3.85%)	11 (21.15%)	**Inf (3.01 – Inf)**	**0.001**
Suicidal tendency	0 (0.00%)	2 (3.85%)	Inf (0.79 – Inf)	0.036
Sleep problems				
No sleep problem	19 (36.54%)	3 (5.77%)	1	
Sleep problem rarely	7 (13.46%)	2 (3.85%)	1.77 (0.12–19.24)	0.612
Sleep problem occasionally	3 (5.77%)	5 (9.62%)	**9.46 (1.17–102.33)**	**0.016**
Sleep problem often	3 (5.77%)	3 (5.77%)	5.79 (0.53–70.71)	0.091
Cannot sleep most nights	4 (7.69%)	3 (5.77%)	4.44 (0.43–48.01)	0.132
Social participation				
No problem with social participation	20 (38.46%)	6 (11.54%)	1	
Avoid social activities as far as possible	11 (21.15%)	8 (15.38%)	2.37 (0.56–10.78)	0.206
Total avoidance of social activities	5 (9.62%)	2 (3.85%)	1.32 (0.10–11.14)	1
Work hours				
> 8 h	13 (25.00%)	7 (13.46%)	1	
5–8 h	3 (5.77%)	2 (3.85%)	1.23 (0.08–13.65)	1
1–4 h	10 (19.23%)	1 (1.92%)	0.19 (0.003–1.94)	0.203
No work	2 (3.85%)	3 (5.77%)	2.67 (0.24–39.28)	0.358
Seasonal work	2 (3.85%)	0 (0.00%)	0 (0.00–11.69)	1
Retired	6 (11.54%)	3 (5.77%)	0.93 (0.11–6.23)	1
Treatment expenditure				
< 40,000 Naira (about US $125)	8 (15.38%)	1 (1.92%)	1	
> 40,000 Naira (about US $125)	21 (40.38%)	14 (26.92%)	5.17 (0.58–252.92)	0.134
Unable to quantify	7 (13.46%)	1 (1.92%)	1.13 (0.01–100.69)	1
Bed net/door and window screens				
No net use	21 (40.38%)	9 (17.31%)	1	
Screen on doors and windows	4 (7.69%)	3 (5.77%)	1.72 (0.21–12.67)	0.659
Bed net use	11 (21.15%)	4 (7.69%)	0.85 (0.16–4.00)	1

Reference category – that with the highest count was chosen as the reference category

Table 5 describes the sources of treatment of study participants. Both cases and controls had multiple sources of treatment/points of contact with either traditional healers, spiritual healers and/or hospital. All controls believed in modern medicine and visited hospitals when ill, whereas 5 cases totally avoided hospital visits and did not believe in conventional medicine, but this difference was not significant (*p* = 0.057). Cases were 10 times more likely to seek health services from either a traditional or spiritual healer (95% CI 3.82–28.85, and 3.52–34.51, respectively).

**Table 5:** Treatment-seeking behaviour of participants

Treatment-seeking behaviours	Control (*n* = 52)	Case (*n* = 52)	*P*-value (Fisher’s test)	OR (95%CI)
No treatment				
Always seeks some form of treatment/remedy ill	52 (100%)	50 (96.15%)	0.49	1.00
Does not always seek treatment/remedy when ill	0 (0%)	2 (3.85%)		Inf (0.19, Inf)
Self-medication				
Frequently attempts to self-medicate	52 (100%)	51 (98.08%)	1	1.00
Rarely attempts to self-medicate	0 (0%)	1 (1.92%)		Inf (0.026, Inf)
Traditional healer				
Does not visit traditional healers when ill	37 (71.15%)	15 (28.84%)	< 0.001	1.00
Visits traditional healers when ill	10 (28.84%)	42 (80.77%)		**10.08 (3.82, 28.85)**
Spiritual healer				
Does not visit spiritual healers when ill	46 (88.46%)	22 (44.31%)	< 0.001	1.00
Visits spiritual healers when ill	6 (11.54%)	30 (57.69%)		**10.19 (3.52, 34.51)**
Hospital				
Does not go to hospital when ill	0 (0%)	5 (9.61%)	0.057	1.00
Goes to hospital when ill	52 (100%)	47 (90.38%)		0 (0.00, 1.05)

### Qualitative analysis

#### Perceptions of stigma

On the perception of stigma between cases and controls, cases were asked if they felt stigmatized whereas controls were asked if they do or will stigmatize persons with lymphedema/hydrocele. A vignette of a lymphedema and hydrocele patient was shown to controls to ascertain if they knew of or had seen anyone with the condition. All controls knew or had come across either a lymphedema or hydrocele patient. Here, 40% of cases reported feeling some sort of stigma from people within and outside their immediate community, and 70% of controls admitted to stigmatizing persons with lymphedema.

Controls were asked if they will be willing to marry if their fiancé/fiancée had lymphedema or hydrocele. Only 27% of controls said they will go ahead with the marriage. Some reasons for this are further highlighted in the responses from the narrative interview (see Additional file 3: Table S3). Cases were asked if their condition affected their relationship or marriage and only 33% of cases reported having relationship/marital problem. At the time of interview, all cases (married and unmarried) were in some form of relationship.

### Narrative interview

**Fig. 2:**
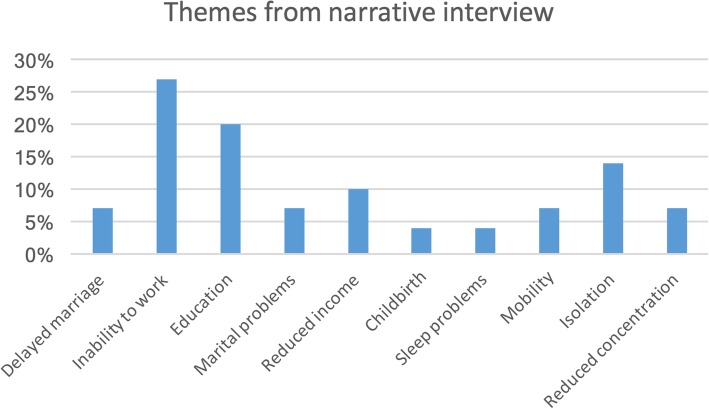
Summary of dominant themes from narrative responses

The narrative responses were grouped into themes according to what the LF patients thought were the main effects of their condition. In Fig. 2, inability to work, inability to continue with their education, reduced income and savings, marital problems and isolation were the most dominant themes.

The following excerpts are from the narrative interviews (see Additional file 4: Table S4 for more quotes):

*“This condition forced me to drop out from school due to the stigma from fellow school mates. I am also isolated. I do not attend social events like wedding ceremonies. I only go to church on Sundays. I do not participate in any extra church activities. It has also depleted my income and savings”.* **Female, 31-40 years. Dreyer stage 6**.

*“I did not have this condition before I got married. I developed this in the last few years. Although my wife has not left me, but the marriage is having a very difficult time. I feel my wife sleeps with other men, because I cannot satisfy her sexually. She comes home late sometimes and does not have any explanation as to why she is home late”.* **Male, >60 years. Dreyer stage 4.**

*“This condition has stunted my educational pursuit. I strongly believe that it is the reason I have not yet gotten married by now. My business is also affected as I cannot function at full capacity again”.* **Male, 31-40 years. Dreyer stage 3.**

*“I have depleted my income and savings of myself and my children in trying to cure my condition”.* **Male, >60 years. Dreyer stage 4.**

## Discussion

The GPELF estimates that elimination of LF as a public health problem and targets to scale down on preventive chemotherapy by 2020 [16, 25]. MDA treatments commenced for endemic LGAs in Anambra State in 2007, with Aguata and Njikoka LGAs starting treatment in 2008 [16]. Although all endemic LGAs have now had more than five [5] rounds of MDA [26], only Plateau and Nasarawa states have shown evidence of interrupting transmission [15]. For the remainder of the country, there has not been any follow-up prevalence survey and thus no evidence of interruption of LF transmission yet. But given that treatment will be scaled down once there is evidence of interruption of transmission, the focus of the LF control programme now needs to shift to include morbidity management. To our knowledge, this is the first study to document chronic LF patients and characterise morbidity in Anambra State, Nigeria.

This study highlights the physical, social and economic impacts of chronic LF, and is in line with findings from in previous research [4, 6, 10, 27–29]. Here we found that chronic LF patients when compared to healthy controls are affected by pain and discomfort, anxiety, reduced mobility, reduced ability to care for self (for instance daily body-washing), and are impaired in their usual domestic and occupational activities. This is an indication that morbidity due to LF alters routine daily activities and incapacitates infected individuals. In many instances, chronic LF patients require constant care and attention, usually from a school-aged member of the family, potentially becoming a burden [9]. This phenomenon has been reported for other similar debilitating diseases like onchocerciasis [23].

From our study, lymphedema and hydrocele patients were more likely to have no formal education. Narrative responses from the qualitative interview cited lymphedema as a major cause of school drop-out, due to inability to carry-on with school work and stigma received from fellow pupils. Thus, LF might have been a contributing factor to the low educational attainment of some cases in this study, and as a result, generally low-income capacity subsequently. However, considering the effect of reverse causality, Cantey et al. reported that low education/literacy level was a risk factor for LF morbidity [30], although we did not explore this further in this work.

Most cases who were able to work only did so for between 1 to 4 h per day. This reduced work day limits their earning capacity, as in many instances income generation is proportional to hours of work. The economic burden of disease is further exacerbated by the cost of treatment as payment is usually out-of-pocket and only 4% of the Nigerian population, mainly federal government employees, are covered by health insurance [31]. Here we show that chronic LF patients spend considerably higher sums of money compared to healthy controls. Economic loss has been previously reported [6, 27, 28], but our findings show much higher estimates for treatment expenditure. Majority of cases spent over 40,000 Naira (about US $125 [32]), which is more than double the government approved monthly minimum living wage of 18,000 Naira (about US $57) [33].

This high cost of treatment is thought to be because chronic LF patients often sought treatment from multiple sources including traditional and spiritual healers, and hospitals [34]. The unavailability of government approved LF morbidity management services may be a driving factor in alternative treatment seeking. Cases were more likely to visit traditional and spiritual healing centres. They may also be more likely to recall and report monies spent on medical treatment. Payment to traditional and spiritual healers were in form of offerings, mainly livestock and agricultural produce. These items are claimed to be used to appease the gods as many perceive disease to be inherited from past generations or caused by witchcraft [7, 8], a phenomenon also reported in studies in other geographical regions [13]. In many instances patients travelled considerable distances and camped for days to weeks in traditional and spiritual healing centres. The cash value of these gift items and monies spent on transportation and lodging are considerably higher than the average treatment cost at most modern medicine practitioners, and are in keeping with findings of studies elsewhere [35]. LF treatment-seeking from alternative medical practitioners may be due to the poor understanding of the general aetiology of disease [7, 8, 12, 13].

The relationship between chronic LF and the mental health of patients is often neglected and insufficiently studied [36]. Here our study found evidence of possible precursors to mental health illness. Lymphedema and hydrocele patients were more prone to social isolation due to stigma, sleep problems, anxiety, problems with concentration and general cognition, and relationship and marital problems; all of which have been reported as potential predictors of depression and mental health illness [37]. Here we have demonstrated that these predictors of mental health problems and depression were more severe in the advanced stages of disease. These findings are in keeping with Obindo et al*’*s study measuring the association of socio-demographic factors and depression in chronic LF patients in Nigeria [38]. Here the researchers diagnosed depression in 20% of survey participants and reported this to be as a result of social isolation, marital problems, literacy level, severity of disease, and financial inadequacy due to impaired functioning. Other studies have also found an association between LF and mental health illness [39, 40].

Although there are specialist mental health facilities available in Nigeria, major treatment gaps exist as there are about 1 trained mental health staff per 100,000 population [41]. ‘Mental health staff’ here includes psychiatrists, nurses, psychologists, social workers, occupational therapists and medical doctors. Health facilities such as psychiatric hospitals are usually located in big cities and urban areas [42], far removed from more rural communities where the majority of chronic LF patients are found. It is thought that this creates a barrier to treatment-seeking as patients would need to travel considerable distance to access care. This is costly in both time and money as patients abandon their income-generating activities and also pay for treatment out-of-pocket, as universal health insurance is not available to the a larger proportion of the Nigerian population [31]. Furthermore, seeking mental healthcare has been found to result in intimidation and stigma from community members [43], thus may serve as a deterrent to seeking care. This highlights some of the challenges of seeking care for mental health illness in Nigeria.

For mental healthcare to be more accessible to those in need, a task-shifting approach may be considered where community health workers and community leaders within the communities are trained to deliver care. This approach has been found to be effective for delivery of mental health services within local communities [44]. This approach also delivers mental health services in a culturally acceptable format.

Here, we demonstrated that chronic LF patients were 72% less likely to use bed nets for protection against mosquito bites. Previous studies showed that interruption of LF transmission was more effective when bed nets were used in synergy with MDA [45–47]. This finding supports the need to integrate bed nets as part of LF control programmes. Mass distribution of bed net has however been implemented in Nigeria under the malaria control programme. Studies investigating the ownership and usage of bed nets demonstrated that although there was a high percentage coverage for bed net ownership, actual usage was considerably lower [48, 49]. This disparity in ownership and usage was associated with level of education, socio-demographic factors and types of housing. It is therefore important to enlighten residents in LF endemic areas of the benefits of bed net usage.

Our study showed that lymphedema and hydrocele cases were more likely to remain unmarried, although majority (67%) of married cases reported not to have problems with their spouses. In contrast, 71% of controls reported unwillingness to marry if their spouse-to-be had lymphedema or hydrocele. This sharp contrast between willingness to remain married to a chronic LF partner and unwillingness to get married to a person with lymphedema/hydrocele may be because married affected persons are economically dependent on each other, already have children in the marriage and thus motivated to remain together and find solutions [23], while unaffected persons see the medical condition as an avoidable problem and are reluctant to take on the burden of caring for a sick partner [23]. These findings are also in line with previous research in the area [50] where exiting a marriage usually does not gain support from family members and may attract stigma from community [50]. This may also be a result of societal and religious views on abandoning a sick partner. This is further highlighted in our qualitative responses where a number of participants interviewed cited marital vows of ‘in sickness and in health’ as their reason for not abandoning a sick partner. The following are some excerpts from the narrative interviews. More quotes are provided in Additional file 2: Table S2.

*“I will not marry even if her behaviour is very good. A person will not see trouble and jump into it. However, if married already, I will not leave her because of the marriage vows”.* **Unaffected male, 51-60 years.**

*“I will not marry if the lady has that condition. Dowry is the same even if your wife is ugly or has a medical condition. If married already, I will not leave my wife if she develops the condition. I will also not like her to leave me if I develop such a medical condition”.* **Unaffected male, 51-60 years**

*“I will not marry because I do not know the treatment and length of time if will take for the disease to be cured and the person to be back to normal. If I was married already and the disease starts to manifest, I will not ask her to leave because we took marriage vows. I will try to find a solution”.* **Unaffected male, 51-60 years**

Although lymphedema and hydrocele patients admitted to feeling stigma, the proportion was in disparity to healthy controls, in which a larger proportion of controls admitted to stigmatising others. This highlights the burden and perception of stigma due to lymphedema and hydrocele in typical rural communities, as patients are more inclined to deny or minimise the feeling of stigma as a mechanism for coping with the condition [23]. Also, as stigma is a sensitive and sometimes emotional discussion for affected persons, some participants in this study were less willing to elucidate on their responses regarding their perception of stigma. This shows that understanding the burden and impact of stigma is complex and a qualitative approach is vital for better understanding of this problem as narrative accounts reveal the nature of concern, fears as well as coping strategies.

Furthermore, hydrocele surgery has been effective in treating patients [51, 52]. It is also an essential component for morbidity management programmes, however, there is inadequate capacity for surgery provision in endemic countries. For this, surgery camps may be established, whereby trained experts hold clinics in endemic communities. Local capacity can also be developed in the management and treatment of hydrocele.

### Study limitations

No sample size calculation was performed for this study. We acknowledge that the limited sample size leads to wide confidence intervals around the reported odds ratios and could potentially lead to biased odds ratios, however, we endeavoured to recruit all lymphedema and hydrocele cases in the study areas. It is impossible to say if this was achieved, but we remained in the field until we were unable to recruit more cases. This study did not ascertain the definite aetiology of lymphedema in cases identified, however it is reasonable to assume that lymphedema in the study area can be attributed to LF due to its high endemicity in the study communities [16]. Podoconiosis is another main cause of lymphedema, however there is no history of this condition or any previously reported cases in the study area. Furthermore, controls were not confirmed to be free from LF as we did not perform any blood confirmatory tests to check for circulating microfilariae, however all controls appeared to be ‘healthy’ with no obvious signs of lymphedema and hydrocele and here we measured psychosocial and economic impacts due to chronic LF, not infection. We also did not attempt to characterise the effects of acute fevers as the intensity and frequency of acute fevers are usually self-reported, therefore prone to reporting bias.

## Conclusions

This study highlights the need for patient-search as a means of estimating the burden of LF morbidity in typical rural settings. Our findings also emphasise the considerable social, physical and economic impacts caused by chronic LF, all of which has adverse effects on the mental health of patients. It is therefore essential to integrate mental health services into morbidity management programmes.

## Additional files

Additional file 1: **Table S1.** Demographics and matching criteria. (DOCX 13 kb)

Additional file 2: **Table S2.** Descriptive statistics of study participants. (DOCX 20 kb)

Additional file 3: **Table S3.** Perception of lymphedema and hydrocele by controls. (DOCX 15 kb)

Additional file 4: **Table S4.** Social impact of lymphedema and hydrocele on cases. (DOCX 17 kb)

## Abbreviations

ADL: Adenolymphangitis
DALY: Disability adjusted life year
EuroQol: European quality of life
GPELF: Global programme for elimination of lymphatic filariasis
LF: Lymphatic filariasis
LGA: Local government Area
mf: Microfilaria
OSD: Onchocercal skin disease
WASH: Water, sanitation and hygiene
