# Supplementary material for: The social, physical and economic impact of lymphedema and hydrocele: a matched cross-sectional study in rural Nigeria
Source: BMC Infect Dis. 2019 Apr 23;19:332. doi: 10.1186/s12879-019-3959-6 (PMC6480436; doi:10.1186/s12879-019-3959-6)
Supplement: Supplementary file 1 — Table S1. Demographics and matching criteria. (DOCX 13 kb) [file 12879_2019_3959_MOESM1_ESM.docx]

S1_Table shows the demographics of matching criteria of study participants. A total 104 participants took part in this survey, of which there were pairs of 16 females and 36 males as cases and controls. This survey was also matched by age and sex.

S1 Table: Demographic characteristics of study participants

| **Demographics** | **Case (N=52)** | **Control (N=52)** |
| --- | --- | --- |
| **Sex** |  |  |
| Male | 36 (34.62%) | 36 (34.62%) |
| Female | 16 (15.38%) | 16 (15.38%) |
| **Age (years of age)** |  |  |
| 21-30 | 3 (2.88%) | 3 (2.88%) |
| 31-40 | 8 (7.69%) | 8 (7.69%) |
| 41-50 | 14 (13.46%) | 14 (13.46%) |
| 51-60 | 12 (11.54%) | 11 (10.58%) |
| >60 | 15 (14.42%) | 16 (15.38%) |
| **LGA** |  |  |
| Aguata | 30 (28.85%) | 28 (26.92%) |
| Njikoka | 22 (21.15%) | 24 (23.08%) |
