# Supplementary material for: The social, physical and economic impact of lymphedema and hydrocele: a matched cross-sectional study in rural Nigeria
Source: BMC Infect Dis. 2019 Apr 23;19:332. doi: 10.1186/s12879-019-3959-6 (PMC6480436; doi:10.1186/s12879-019-3959-6)
Supplement: Supplementary file 2 — Table S2. Descriptive statistics of study participants. (DOCX 20 kb) [file 12879_2019_3959_MOESM2_ESM.docx]

In S2_Table, 56% of females were between age 41-50, whereas males where generally older with 64% being at least 51 years of age. A total of 24 cases were classified as Dreyer stage 1-3, and 4 males had hydrocele. The majority of cases (>60%) reported that they had no problem with self-care (35/52), usual activity (38/52), and cognition (46/52). Half of the cases did not have problems with social participation. However, cases reported moderate to severe problems for pain/discomfort, pain frequency, pain severity, and anxiety. Considerably more cases spent over US $125 on their last health visit. 94% and 75% of females sought treatment from a traditional healer and spiritual healer respectively, as compared to 75% and 50% of males. 58% of total cases did not use bed nets or door and window screens.

S2_Table: Descriptive statistics of LF cases according to sex

| **Characteristics** | **Female (N=16)** | **Male (N=36)** |
| --- | --- | --- |
| **Age group (Years)** |  |  |
| 21-30 | 1 (6.25%) | 2 (5.55%) |
| 31-40 | 2 (12.5%) | 6 (33.33%) |
| 41-50 | 9 (56.25%) | 5 (13.9%) |
| 51-60 | 1 (6.25%) | 10 27.77%) |
| >60 | 3 (18.75%) | 13 (36.11%) |
| **LGA** |  |  |
| Aguata | 8 (50%) | 22 (61.11%) |
| Njikoka | 8 (50%) | 14 (38.9%) |
| **Marital status** |  |  |
| Married | 11 (69.75%) | 29 (80.56%) |
| Divorced | 0 (0%) | 2 (5.56%) |
| Never married | 5 (31.25%) | 5 (13.89%) |
| **Occupational level** |  |  |
| No work | 3 (18.75%) | 2 (5.56%) |
| Unskilled | 10 (62.5%) | 34 (94.44%) |
| Skilled | 3 (18.75) | 0 (0%) |
| **Dreyer Stage** |  |  |
| Stage 1-3 | 11 (68.75%) | 13 (36.11%) |
| Stage 4-5 | 0 (0%) | 12 (33.33%) |
| Stage 6-7 | 5 (31.25%) | 7 (19.44%) |
| Hydrocele | N/A | 4 (11.11%) |
| **Educational level** |  |  |
| No formal education | 3 (18.75%) | 6 (16.67%) |
| Primary education | 8 (50%) | 18 (50%) |
| Secondary education | 4 (25%) | 9 (25%) |
| Tertiary education | 1 (6.25%) | 3 (8.33%) |
| **Mobility** |  |  |
| No problem | 8 (50%) | 12 (33.33%) |
| Mild problem | 5 (31.25%) | 8 (22.22%) |
| Moderate problem | 0 (0%) | 3 (8.33%) |
| Severe problem | 3 (18.75%) | 13 (36.11%) |
| **Self-care** |  |  |
| No problem | 13 (81.25%) | 22 (61.11%) |
| Difficulty in self-care | 2 (12.5%) | 5 (13.89%) |
| Only essential needs are met | 0 (0%) | 3 (8.33%) |
| Require someone to help for care | 0 (0%) | 6 (16.67%) |
| Unable to take care of self | 1 (6.25%) | 0 (0%) |
| **Usual activity** |  |  |
| No problem | 13 (81.25%) | 25 (69.44%) |
| Usual activity performed with difficulty | 2 (12.5%) | 7 (19.44%) |
| Only essential activity performed | 1 (6.25%) | 3 (8.33%) |
| No activity at all | 0 (0%) | 1 (2.78%) |
| **Pain and discomfort** |  |  |
| No pain | 0 (0%) | 1 (2.78%) |
| Mild pain, without Self-treatment | 11 (68.75%) | 9 (25%) |
| Mild pain, with Self-treatment | 0 (0%) | 7 (19.44%) |
| Compelled to rest | 5 (31.25%) | 19 (52.78%) |
| **Pain frequency** |  |  |
| No pain | 0 (0%) | 1 (2.78%) |
| Weekly | 2 (12.5%) | 7 (19.44%) |
| Monthly | 12 (75%) | 12 (33.33%) |
| Daily | 2 (12.5%) | 12 (33.33%) |
| Constant | 0 (0%) | 4 (11.11%) |
| **Pain severity** |  |  |
| No pain | 0 (0%) | 1 (2.77%) |
| Mild | 0 (0%) | 3 (8.33%) |
| Medium | 4 (25%) | 14 (38.9%) |
| Severe | 12 (75%) | 18 (50%) |
| **Cognition** |  |  |
| No problem | 14 (87.5%) | 32 (88.89%) |
| Reduced concentration, memory affected | 2 (12.5%) | 2 (5.56%) |
| Loss of concentration | 0 (0%) | 2 (5.56%) |
| **Anxiety** |  |  |
| No anxiety | 0 (0%) | 6 (16.67%) |
| Does not interfere with performance | 3 (18.75%) | 6 (16.67%) |
| Leads to low performance and irritating tendency | 9 (56.25%) | 13 (36.11%) |
| No performance and total detachment | 4 (25%) | 9 (25%) |
| Suicidal tendency | 0 (0%) | 2 (5.56%) |
| **Sleep problems** |  |  |
| No sleep problem | 10 (62.5%) | 12 (33.33%) |
| Sleep problems rarely | 3 (18.75%) | 6 (16.67%) |
| Sleep problems occasionally | 2 (12.5%) | 6 (16.67%) |
| Sleep problems often | 0 (0%) | 6 (16.67%) |
| Can’t sleep most nights | 1 (6.25%) | 6 (16.67%) |
| **Social participation** |  |  |
| No problems with social participation | 12 (75%) | 14 (38.89%) |
| Avoid social activities as far as possible | 1 (6.25%) | 18 (50%) |
| Total avoidance of social activities | 3 (18.75%) | 4 (11.11%) |
| **Work hours** |  |  |
| >8 hours | 4 (25%) | 16 (44.44%) |
| 5-8 hours | 2 (12.5%) | 3 (8.33%) |
| 1-4 hours | 6 (37.5%) | 5 (13.89%) |
| No work | 0 (0%) | 5 (13.89%) |
| Seasonal work | 4 (25%) | 5 (13.89%) |
| Retired | 0 (0%) | 2 (5.56%) |
| **Treatment-seeking behaviour** |  |  |
| **No treatment** |  |  |
| Always seeks some form of treatment/remedy ill | 16 (100%) | 34 (94.44%) |
| Does not always seek treatment/remedy when ill | 0 (0%) | 2 (5.56%) |
| **Self-medication** |  |  |
| Frequently attempts to self-medicate | 16 (100%) | 35 (97.22%) |
| Rarely attempts to self-medicate | 0 (0%) | 1 (2.77%) |
| **Traditional healer** |  |  |
| Does not visit traditional healers when ill | 1 (6.25%) | 9 (25%) |
| Visits traditional healers when ill | 15(93.75%) | 27 (75%) |
| **Spiritual healer** |  |  |
| Does not visit spiritual healers when ill | 4 (25%) | 18 (50%) |
| Visits spiritual healers when ill | 12 (75%) | 18 (50%) |
| **Hospital** |  |  |
| Does not go to hospital when ill | 0 (0%) | 5 (31.25%) |
| Goes to hospital when ill | 16 (100%) | 31 (68.75%) |
| **Treatment expenditure on last hospital visit** |  |  |
| <40,000 Naira (about US $125) | 2 (12.5%) | 7 (19.44%) |
| >40,000 Naira (about US $125) | 9 (56.25%) | 26 (72.22%) |
| Unable to quantify | 5 (31.25%) | 3 (8.33%) |
| **Bed net/door and window screens** |  |  |
| No net use | 11 (68.75%) | 19 (52.78%) |
| Screen on doors and windows | 2 (12.5%) | 5 (13.89%) |
| Bed net use | 3 (18.75%) | 12 (33.33%) |
