# Supplementary material for: The social, physical and economic impact of lymphedema and hydrocele: a matched cross-sectional study in rural Nigeria
Source: BMC Infect Dis. 2019 Apr 23;19:332. doi: 10.1186/s12879-019-3959-6 (PMC6480436; doi:10.1186/s12879-019-3959-6)
Supplement: Supplementary file 3 — Table S3. Perception of lymphedema and hydrocele by controls. (DOCX 15 kb) [file 12879_2019_3959_MOESM3_ESM.docx]

**S3_Table: Qualitative responses of perception of lymphedema and hydrocele by controls**

|  | **Will you discriminate against someone with lymphedema or hydrocele?** | **Will you be willing to marry if you discover that your husband/wife to be has lymphedema or hydrocele?** |
| --- | --- | --- |
| Female 51-60 | *If it was a relative, I will not discriminate, but if it is a stranger, I will discriminate and stay away from the person.* | *I will not marry if my husband to be has bad leg* |
| Female 31-40 | *I do not discriminate.* | *I will not marry if my husband-to-be has elephantiasis.* |
| Female 31-40 | *I will not discriminate if the person is related to me. But if the person is an outsider, I will not accept them fully.* | *I will not marry if my man has bad leg.* |
| Female 31-40 | *I will not stigmatize if I see anyone with elephantiasis.* | *I will not marry if my husband has the condition.* |
| Female 21-30 | *I do not stigmatize.* | *I will not marry if my husband has swollen leg.* |
| Female 51-60 | *I do not discriminate against them.* | *I will marry if the condition is manageable. Nothing is impossible before God.* |
| Female 21-30 | *I will not discriminate unless it is oozing fluid and smelly.* | *I will marry not minding the condition.* |
| Male >60 | *I do not discriminate. They are my brothers and sisters. I cannot discriminate against them.* | *I will still marry her even of her leg is swollen. The most important thing in marriage is the behaviour of your spouse.* |
| Male 51-60 | *I do not discriminate against them. I know people with this condition. We attend meetings together. I am also the community-directed distributor (CDD) of Ivermectin in my community.* | *I will not marry because I do not know the treatment and length of time if will take for the disease to be cured and the person to be back to normal. If I was married already and the disease starts to manifest, I will not ask her to leave because we took marriage vows. I will try to find a solution.* |
| Female 51-60 | *I do not discriminate. The lady I buy bananas from has this condition, and I still patronize her.* | *I will not marry if his leg is swollen. If married already, I will not leave him if he develops the ailment because he will not leave me if I develop swollen leg.* |
| Male 51-60 | *I do not discriminate.* | *I will not marry even if her behaviour is very good. A person will not see trouble and jump into it. However, if married already, I will not leave her because of the marriage vows.* |
| Male 51-60 | *It is irritating. Yes, I discriminate and avoid them. I give sufferers a safe distance.* | *I will not marry if the lady has that condition. Dowry is the same even if your wife is ugly or has a medical condition. If married already, I will not leave my wife if she develops the condition. I will also not like her to leave me if I develop such a medical condition.* |
| Male >60 | *I do not stigmatize. There is no point to avoid people with that kind of condition.* | *I will marry her even if her leg is swollen. It all depends on the love we have for each other. I will not leave my wife because of a medical problem.* |
| Male 41-50 | *I stigmatize. Yes, I feel it is infectious and contagious. So I try to protect myself by staying away and far from sufferers of this condition.* | *I will not marry if the leg is big or swollen. I will not divorce her due to her condition. Marriage vows say for better for worse, in sickness and in health, till death do us part.* |
| Male >60 | *I avoid them. I think it is infectious, so I stay away.* | *I will not marry if the lady has a bad leg. I will not also leave her if we were already married before she develops the problem.* |
| Male 51-60 | *I have not yet seen anyone with this condition, but I believe that I will discriminate if I come across any.* | *I will also not marry if I know my wife has this condition. But I will not divorce her if we were married already and she develops this medical condition.* |
| Female 21-30 | *I do not discriminate. They are human beings like me.* | *My marrying him depends on the extent of my love for him.* |
| Female 31-40 | *No, I do not stigmatize.* | *People are not born with this condition. It is not their fault that they have elephantiasis. Yes, I will marry him if I truly love him. In fact this is the ultimate test for love.* |
| Female >60 | *It is not infectious. I do not stigmatize.* | *I will demand he treats himself first. But if he was born with the deformity, I can now confirm that it is not his fault, therefore I will marry him.* |
| Female 21-30 | *It is not in my character to discriminate.* | *If I love him, I will marry him no matter the condition.* |
| Female 51-60 | *I do not stigmatize. In fact I have a neighbour who has elephantiasis and we are very friendly.* | *I will not answer that question.* |
