# Supplementary material for: The social, physical and economic impact of lymphedema and hydrocele: a matched cross-sectional study in rural Nigeria
Source: BMC Infect Dis. 2019 Apr 23;19:332. doi: 10.1186/s12879-019-3959-6 (PMC6480436; doi:10.1186/s12879-019-3959-6)
Supplement: Supplementary file 4 — Table S4. Social impact of lymphedema and hydrocele on cases. (DOCX 17 kb) [file 12879_2019_3959_MOESM4_ESM.docx]

**S4_Table: Qualitative responses of the social impact of lymphedema and hydrocele on cases**

|  | **Impact of disease on life in general** | **Do you experience stigmatization?** | **Impact of disease on your marriage and relationship** |
| --- | --- | --- | --- |
| Female, 41-50  Dreyer stage 3 | *I have been engaged for 3 years. My fiancé is waiting for me to get well before we get officially married.* | *I do not feel stigma* | *This condition has delayed my wedding as I want to look beautiful and at my best on my wedding day.* |
| Male, >60  Hydrocele | *I have slowed down with work, because if I work so hard, it gets painful. I do not engage sexually with my wife anymore. I also feel ashamed so I do not socialize much again.* | *I feel stigma* | *My wife is poor so cannot help financially. However, she tolerates me. But sometimes she becomes very rude. I believe she is still in the marriage because we have children together.* |
| Male, 31-40  Dreyer stage 3 | *This condition has stunted my educational pursuit. I strongly believe that it is the reason I have not yet gotten married by now. My business is also affected as I cannot function at full capacity again.* | *I do not feel stigma from people I know. I cannot tell about others.* | *I am not yet married and feel this condition is the reason I am still single.* |
| Male, >60  Dreyer stage 4 | *It has prevented me from participating in football which is my usual hubby. I cannot pay school fees for my child in the university because I am unable to work and earn money* | *I attend village meetings and I do not feel stigma from people in these meetings.* | *My wife is supportive.* |
| Male, 31-40  Dreyer stage 4 | *I am unable to work whenever the acute fever sets in.* | *I do not feel stigma.* | *I even had the condition before I got married. My wife loves me and is very supportive.* |
| Male 51-60,  Hydrocele | *I plan to get married in 2 months (from the date of this interview). I have not disclosed my condition to my fiancée. I am afraid at what her reaction would be.* | *People do not know of my condition because it is always hidden away in my trousers. I cannot tell if I will be stigmatized against. However, I think strangers will distance themselves from me.* | *My relationship has no problems because my fiancé does not know about my condition. .* |
| Male >60,  Hydrocele | *It has affected my relationship with my wife. This has also affected my ability to work, although work is not daily, but my capacity to function is now restricted.* | *I feel stigmatized against. When I come into gatherings, other people usually say I should go out.* | *I did not have this condition before I got married. I developed this in the last few years. Although my wife has not left me, but the marriage is having a very difficult time. I feel my wife sleeps with other men, because I cannot satisfy her sexually. She comes home late sometimes and does not have any explanation as to why she is home late.* |
| Male >60,  Dreyer stage 4 | *I have depleted my income and savings of myself and my children in trying to cure my condition.* | *I keep it clean. I do not feel stigma.* | *My wife is supportive.* |
| Female, 41-50  Stage 3 | *This condition has stunted my progress in life. It made me drop out from primary school and caused late marriage.* | *I do not feel stigma. I run a restaurant and people still came to my stall to eat and relax.* | *It delayed/affected my chances of getting married. However, my husband married me not minding my swollen leg. After we got married, he did not help in finding a solution because he has no money.* |
| Female, 31-40 Dreyer stage 3 | *I had three miscarriages and I believe it is due to my condition.* | *I do not feel stigma from people.* | *My husband is very supportive both emotionally and financially.* |
| Male >60,  Dreyer stage 4 | *My wife left me because of my swollen leg. We have no children together.* | *I keep it neat, so people do not stigmatize against me. But some people still stigmatize.* | *My marriage broke down when my leg problem started. My wife left me.* |
| Female 21-30,  Dreyer stage 3 | *We have spent plenty of money trying to find a remedy for this leg.* | *I do not feel stigmatization.* | *I feel that it is because of this that I have not found a husband.* |
| Male >60,  Dreyer stage 4 | *This condition affects my sleep too much. I cannot sleep with the hotness from the fever sets in. This condition has also made me quit my job.* | *I do not feel stigma. One of the reasons for not feeling stigma is because community members know about this condition and what causes it. They know is it not as a result of dirtiness and that sufferers where not born with it.* | *I do not have a problem with my marriage.* |
| Male 41-50  Dreyer stage 6 | *I walk with great difficulty. I now live a solitary life. I do not go out of my compound.* | *I do not feel stigma.* | *I do not have problems with my marriage.* |
| Female 31-40,  Dreyer stage 6 | *This condition forced me to drop out from school due to the stigma from fellow school mates. I am also isolated. I do not attend social events like wedding ceremonies. I only go to church on Sundays. I do not participate in any extra church activities. It has also depleted my income and savings.* | *I feel severe stigma.* | *This is also the reason why I did not get married. Now that I am 39 years, it will be very difficult for me to still get married.* |
| Male 51-60,  Dreyer stage 4 | *This condition does NOT affect my business.* | *I do not feel stigma now because the leg is clean. But when it was smelly, people used to stay away from me.* | *I do not have a problem with my marriage. My wife is supportive.* |
| Male 31-40,  Dreyer stage 6 | *This condition reduces my concentration during conversations. It affects my ability to learn bricklaying. It also affects full participation in social activities.* | *I feel severe stigma.* | *Although I am now married, it was very difficult because ladies avoid me when they learn of my condition. Now I am married, my wife loves and supports me.* |
| Male 41-50,  Dreyer stage 6 | *I avoid social engagements as much as I can. I restrict my participation. I am also unable to continue my farm work and pay school fees for my children.* | *I feel stigma. If I get into public transport, other passengers disembarked from the vehicle.* | *This has not affected my marriage.* |
| Female >60,  Dreyer stage 3 | *Mobility is exacerbated due to arthritis in my knees. I have spent all my pension and gratuity in trying to find a remedy for this condition.* | *I do not feel stigma.* | *This does not affect my marriage.* |
| Male 21-30,  Dreyer stage 3 | *I dropped out of school due to the severity of this disease. I went back to school after 3 years when I got over the initial shock and after encouragement from friends and family members.* | *I do not feel stigma.* | *This does not affect my relationship with the opposite sex. My girlfriend does not mind.* |
| Male >60,  Dreyer stage 6 | *I have stop my goat selling business because of my condition. My money has also finished.* | *I keep the affected area clean. I do not feel stigma.* | *My wife married me not minding my condition. She has lived and supported me ever since we got married.* |
